# YAP1/TAZ Mediates Rumen Epithelial Cell Proliferation but Not Short-Chain Fatty Acid Metabolism In Vitro

**DOI:** 10.3390/ani14060922

**Published:** 2024-03-17

**Authors:** Bin Yang, Zebang Xu, Hongwei Chen, Tingting Ma, Yiming Zhao, Mengxin Pang, Jiakun Wang

**Affiliations:** 1 School of Biological and Chemical Engineering, Zhejiang University of Science and Technology, Hangzhou 310023, China; binyang@zust.edu.cn (B.Y.); 17858528377@163.com (T.M.); 13320717081@163.com (Y.Z.); 19550179660@163.com (M.P.); 2 Institute of Dairy Science, College of Animal Sciences, Zhejiang University, Hangzhou 310058, China; 22017023@zju.edu.cn (Z.X.); chenhwme@zju.edu.cn (H.C.); 3 Key Laboratory of Molecular Animal Nutrition (Zhejiang University), Ministry of Education, Hangzhou 310058, China

**Keywords:** rumen epithelial cell, proliferation, YAP1/TAZ, GA-017, verteporfin, sodium butyrate

## Abstract

**Simple Summary:**

Rumen development is important to the health and performance of ruminants. The proteins YAP1 and TAZ are key regulators of the mammalian epithelium. However, it is unclear whether YAP1/TAZ mediates the development of rumen epithelium. Therefore, we assessed the effects of YAP1/TAZ on rumen epithelial cell proliferation. Sodium butyrate is a nutrient that can be used to promote rumen development. We also assessed whether sodium butyrate mediated rumen epithelial development through the activation of YAP1/TAZ. The results indicated that YAP1/TAZ activation could promote rumen epithelial cell proliferation but not short-chain fatty acid metabolism, and sodium butyrate did not function in a YAP1/TAZ-dependent manner.

**Abstract:**

Promoting rumen development is closely related to the health and efficient growth of ruminants. The transcriptional co-activators Yes1-associated protein (YAP1) and WW domain-containing transcription regulator protein 1 (TAZ) are key regulators of the mammalian epithelium. In the present study, we assessed the impact of YAP1/TAZ on rumen epithelial (RE) cell proliferation using their activator GA-017 (GA) and inhibitor verteporfin (VP). We also investigated whether YAP1/TAZ-dependent alteration was involved in the RE developmental process induced by sodium butyrate (SB). The results indicated that GA promoted RE cell proliferation, while VP disrupted RE cell proliferation. The Hippo, Wnt, and calcium signaling pathways were altered following the regulation of YAP1/TAZ. Upon YAP1/TAZ activation, the expression of *CCN1*/*2* increased. However, when YAP1/TAZ was inhibited, the expression of *BIRC3* decreased. In the SB-treated cells, YAP1/TAZ-induced changes were not observed. SB increased the expressions of differentiated cell marker genes and genes involved in short-chain fatty acid (SCFA) metabolism, while YAP1/TAZ did not. Thus, YAP1/TAZ could be potential targets for regulating RE cell proliferation but not for SCFA metabolism. SB could not affect YAP1/TAZ. These findings broaden our understanding of the role of YAP1/TAZ and their regulators in RE development.

## 1. Introduction

The global challenge of food security can undermine the world’s capacity to meet its current and future food needs [1]. As an important source of high-quality animal protein, promoting the health and efficient growth of ruminants will help to improve the sustainability of food systems. Rumen is a key digestive organ that constitutes more than 70% of the total volume of the digestive tract in ruminants [2]. Compared to the intestine or the stomach of monogastric animals, the rumen is a unique organ that is covered by a stratified squamous epithelium, including the stratum corneum, granulosum, spinosum, and basal layer [3]. The rumen epithelium (RE) plays a key role in the absorption and metabolism of short-chain fatty acids (SCFAs). In addition, the rumen is an efficient organ that provides the animal host with up to 70% of its daily energy in the form of ketone bodies (mainly β-hydroxybutyrate) [4]. In neonates, the rumen is underdeveloped, small in size, and essentially nonfunctional in terms of metabolic capacity [5]. Because rumen development is closely associated with slaughter performance and milk yield [6,7], promoting cell proliferation and stimulating the metabolic capacity of RE are vital for the health and efficient growth of ruminants.

The transcriptional co-activators Yes1-associated protein (YAP1) and WW domain-containing transcription regulator protein 1 (WWTR1, also known as TAZ) are fundamentally important regulators of stem cell proliferation, tissue regeneration, and tumor formation in mammalian epithelia [8,9,10]. The activation or overexpression of YAP1/TAZ is positively associated with the proliferative capacity of stratified squamous epithelium [10,11]. It is unclear whether YAP1/TAZ mediates RE cell proliferation.

GA-017 (GA) and verteporfin (VP) are identified as efficient activators and inhibitors for YAP1/TAZ, respectively. GA can activate YAP1/TAZ by inhibiting their phosphorylation and promoting their nuclear translocation [12]. VP disrupts YAP1/TAZ-TEA domain transcription factor (TEAD)-mediated transcription and suppresses downstream gene expression [9]. GA and VP are commonly used to investigate the functions of YAP1/TAZ in various developmental processes or tumor progressions, such as the proliferation of ovarian cancer cells [12], the reprogramming of colonic epithelium [13], and the homeostasis and repair of skin epithelium [14]. In addition, VP has been successfully used to inhibit YAP1 activity in bovine preovulatory granulosa cells [15]. Thus, GA and VP could be proper tools for studying the function of YAP1/TAZ in RE cell proliferation.

Sodium butyrate (SB) has been certified to improve animal performance and rumen papillae growth at a morphological level [16] and ameliorate high-concentrate-diet-induced inflammation in the rumen [17]. At the gene expression level, SB infusion upregulated the mRNA expression of cyclins and genes involved in SCFA uptake and metabolism and downregulated the mRNA expression of apoptosis-related genes in RE to promote ruminal papillae growth and SCFA metabolic capacity [18]. It is unknown whether the YAP1/TAZ-dependent change is involved in the RE development induced by SB.

To fill these knowledge gaps, we aimed to assess the impact of YAP1/TAZ on RE proliferation. The transcriptomic expression was analyzed to investigate the potential regulatory networks. We also investigated whether the YAP1/TAZ-dependent change is involved in the SB-induced RE cell proliferation process. Our results will provide new insights for promoting rumen development and precise nutrition in growing ruminants.

## 2. Materials and Methods

### 2.1. Ethics Statement

The experimental protocols were approved by the Animal Care Committee of Zhejiang University (Hangzhou, China, ZJU17234), and our experimental procedures adhered to the guidelines for animal research of Zhejiang University.

### 2.2. Experimental Design

Two in vitro experiments were conducted in the current study. In Experiment 1, we investigated the effects of YAP1/TAZ on the transcriptomic expression of RE cells. The RE cells were treated with either GA (GA group) or VP (VP group) or left untreated (Con group) to assess gene expression. In Experiment 2, we investigated whether a YAP1/TAZ-dependent change was involved in the SB-induced RE developmental process. The RE cells were treated with SB (SB group) or left untreated (Con group) to assess gene expression. Cells were seeded in 6-well plates at a density of 2000 cells/100 µL. The cell experiment was conducted in triplicate wells. After the experiment, cells were harvested and stored at −80 °C for transcriptomic analysis.

### 2.3. Cell Culture

The primary RE cells used in the present study were previously isolated from the RE tissue of newborn Hu lambs by our research group. The isolation and culture methods were performed according to previous studies with some modifications [19,20]. In brief, the cut RE tissues (1 cm^2^) were washed with an ice-cold D-Hank’s balanced salt solution containing 500 U/mL penicillin, 500 μg/mL streptomycin, 100 μg/mL gentamycin, and 5 μg/mL amphotericin B several times until the solution was free of any contaminants. The tissue was digested with a 0.25% trypsin–EDTA solution (Solarbio, Beijing, China) at 37 °C for 30 min, and the resulting solution was then discarded. Afterward, the RE tissue was digested once more with a 0.25% trypsin–EDTA solution for 1.5 to 2 h. Every 10 min, the digestion solution was collected and replaced with a fresh solution. The collected solution was centrifuged at 300× *g* for 5 min at 4 °C to collect cell pellets. Cells were cultured in DMEM containing 2% FBS, 50 U/mL penicillin, 50 μg/mL streptomycin, and 1% mixed additive (including 25 ng/mL epidermal growth factor, 100 ng/mL hydrocortisone, 10 μg/mL insulin, 5 μg/mL transferrin, 87 ng/mL cholera toxin, and 1.3 × 10^–2^ ng/mL triiodothyronine) and incubated at 37 °C with 5% CO_2_.

### 2.4. Optimal Treatment Condition Selection for GA, VP, and SB

The GA and VP were diluted with DMSO to different concentrations (0, 1, 2.5, 5, 10, and 20 µM for GA and 0, 0.5, 1, 2.5, and 5 µM for VP). The final concentration of DMSO was less than 1‰ (*v*/*v*) in the treatment solution. The SB was diluted with ddH_2_O to concentrations of 0, 0.25, 0.5, 1, 2.5, 5, 10, 25, and 50 µM. RE cells were cultured with various concentrations of GA, VP, or SB for 4, 8, 12, 24, and 48 h to determine the optimal concentration and culture duration for each solution based on cell viability.

### 2.5. Cell Viability Assay

Cell Counting Kit-8 (Beyotime, Shanghai, China) was used to measure the viability of RE cells following the manufacturer’s instructions. The cells were counted in duplicate wells. An automated microplate reader (Thermo Scientific, USA) was used to measure the absorbance at 450 nm. The cell viability was calculated using the formula (OD_treatment_ − OD_blank_)/(OD_control_ − OD_blank_) × 100%, where OD represents optical density.

### 2.6. RNA Extraction, Sequencing, and Raw Data Processing

Total RNA was extracted from RE cells using a total RNA extraction kit (Aidllab, Beijing, China) following the manufacturer’s instructions. A Nanodrop 2000 spectrophotometer (Thermo Scientific, Wilmington, DE, USA) was used to measure the concentration and purity of the extracted RNA. The RNA Nano 6000 Assay Kit and Bioanalyzer 2100 system (Agilent Technologies, Santa Clara, CA, USA) were used to measure RNA integrity. The integrity of the RNA samples used in the present study was greater than 8.5. A total of 3 μg of RNA per sample was used to generate a cDNA library using the NEBNext UltraTM RNA Library Prep Kit for Illumina^®^ (NEB, Beverly, MA, USA) following the manufacturer’s instructions. The library fragments were then purified and amplified. The Agilent Bioanalyzer 2100 system was used to evaluate the quality of the library. The libraries were then sequenced on an Illumina HiSeq 4000 to generate 150 bp paired-end reads by Novogene Bioinformatics Technology Co., Ltd. (Tianjin, China).

Quality control was performed on the raw data by removing reads containing adapters or poly-N as well as low-quality reads using CASAVA (version 1.8, Illumina, San Diego, CA, USA). The clean data were mapped to the sheep genome (Oar_rambouillet_v1.0) using HISAT2 (version 2.2.1) [21].

### 2.7. Gene Expression Analysis

Fragments per kilobase of exon model per million mapped fragments (FPKM) were calculated to represent the mRNA expression levels. Genes with FPKM ≥ 1 in all three samples of one group were considered to be expressed genes and were subjected to downstream analysis. To generate a relationship between expressed genes and cell viability, we conducted weighted correlation network analysis (WGCNA) using R software [22]. The differentially expressed gene (DEG) analysis between the treatment group and Con was conducted using DESeq2 [23] in R software (v3.6.3). Genes with a false discovery rate (*FDR*) < 0.05 and a |log_2_fold change| > 1 were considered DEGs.

### 2.8. Quantitative PCR (qPCR) Analysis

A ReverTra Ace qPCR RT Kit (Toyobo, Osaka, Japan) was used to perform the reverse transcription. For each reaction, 1 μg of total RNA was used in 20 μL of the total reaction volume according to the manufacturer’s instructions. Primers used in the present study were designed using the Primer-BLAST tool in the Basic Local Alignment Search Tool (BLAST) of the National Center for Biotechnology Information (NCBI) (Table S1). The amplification products were sequenced and searched using BLAST to validate the specificity of these primers. The qPCR was performed with a 20 μL reaction volume using FastStart Universal SYBR Green Master (Roche, Basel, Switzerland) on the ABI 7500 Real-Time PCR system (Applied Biosystems Inc., Foster City, CA, USA). The relative mRNA expression levels were normalized to the expression of *GAPDH* [24] using 2^−(Ct of target genes−Ct of *GAPDH*)^.

### 2.9. Function Enrichment Analysis

Genes were enriched for Gene Ontology (GO) and Kyoto Encyclopedia of Genes and Genomes (KEGG) using the “clusterProfiler” package in R [25]. In the GO enrichment analysis, only “Biological Process” GO terms were considered. The GO term and KEGG pathway were considered significant with a *p*-value < 0.05 and a gene count > 2.

### 2.10. Statistical Analysis

A one-way ANOVA was used to analyze the concentration effect of GA, VP, and SB using IBM SPSS Statistics 25 software (IBM Corp., Armonk, NY, USA). Dunnett’s test was conducted to compare the means of different concentration groups against the mean of the Con group. Significance was defined as *p* < 0.05.

## 3. Results

### 3.1. Optimal Treatment Conditions for GA and VP on the Viability of RE Cells

**Figure 1:**
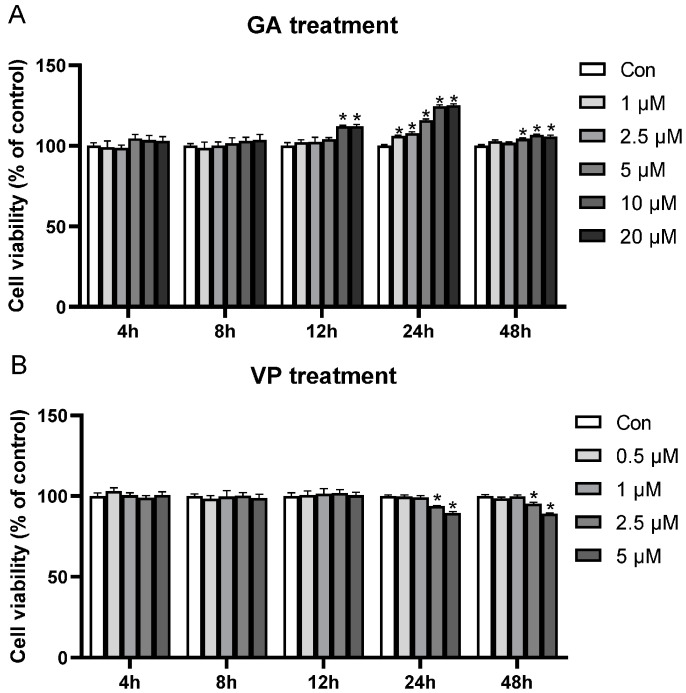
Optimal treatment conditions for GA-017 (GA) and verteporfin (VP) for regulating the viability of rumen epithelial (RE) cells. (**A**) The effect of different concentrations of GA (0, 1, 2.5, 5, 10, and 20 µM) on the viability of RE cells at different incubation times (4, 8, 12, 24, and 48 h). (**B**) The effect of different concentrations of VP (0, 0.5, 1, 2.5, and 5 µM) on the viability of RE cells at different incubation times (4, 8, 12, 24, and 48 h). * represents *p* < 0.05 when compared to control group.

To determine the optimal concentration and incubation time of YAP1/TAZ activator GA and inhibitor VP, we studied the effects of varying GA and VP concentrations on the viability of RE cells at different incubation times. Compared to Con, cell viability significantly increased when treated with 10 and 20 μM GA for 12 h; 1, 2.5, 5, 10, and 20 μM GA for 24 h; and 5, 10, and 20 μM GA for 48 h (*p* < 0.05, Figure 1A). The highest cell viability was observed when cells were treated with 10 and 20 μM GA (Figure 1A). The cell viability was not affected when treated with different concentrations of GA for 4 or 8 h (*p* > 0.05, Figure 1A). On the other hand, cell viability significantly decreased when treated with 2.5 and 5 μM VP for 24 or 48 h, compared to Con (*p* < 0.05, Figure 1B). No significant effect of VP was observed when cells were incubated for 4, 8, or 12 h (*p* > 0.05, Figure 1B). Thus, 10 μM GA and 5 μM VP with an incubation time of 24 h were chosen for Experiment 1.

### 3.2. Transcriptomic Profiles of RE Cells Treated with GA and VP

In Experiment 1, after filtering out the low-quality reads, 378.81 million high-quality reads were obtained across 9 samples, with an average of 42.09 ± 1.12 (mean ± SD) million reads per sample (Table S2). Over 85.02% of the clean reads for each sample were mapped to the reference genome. In total, 13,390 genes were identified as expressed genes and were subjected to downstream analysis.

**Figure 2:**
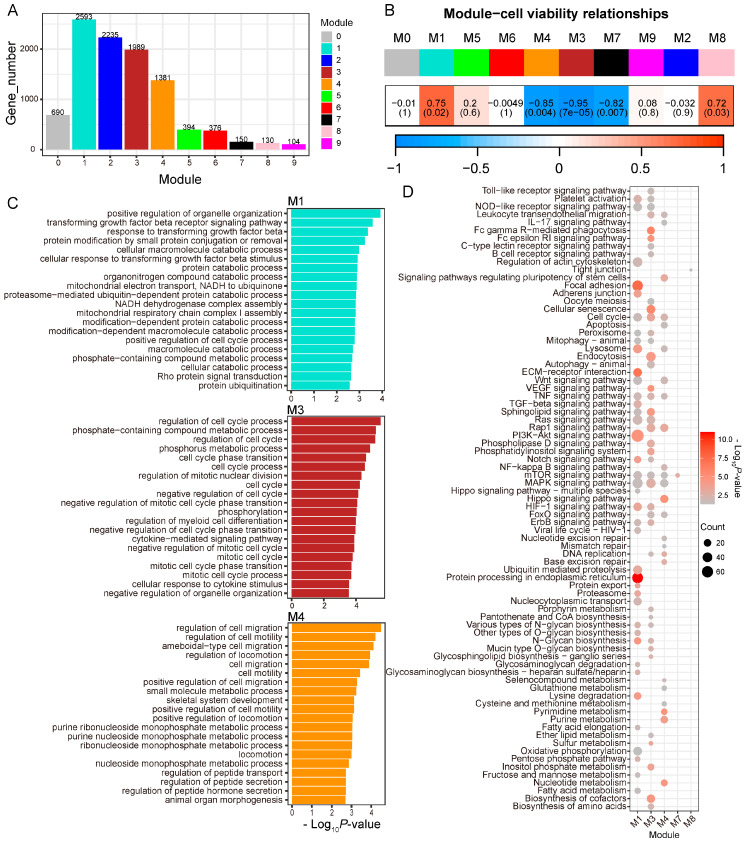
The relationship between gene co-expression networks and cell viability. (**A**) The number of genes in each module generated by WGCNA. (**B**) Pearson correlation between modules and cell viability. (**C**) Top 20 GO terms enriched by modules (M) 1, 3, and 4. (**D**) KEGG pathways enriched by M1, 3, 4, 7, and 8.

The expressed genes were clustered into 10 gene modules using WGCNA. Among them, Module 1 (M1) contained the highest number of genes, followed by M2, M3, and M4 (Figure 2A). To determine the relationship between gene modules and cell viability, we correlated gene modules with cell viability. M1 and M8 showed a positive correlation with cell viability, while M3, M4, and M7 exhibited a negative correlation with cell viability (*p* < 0.05, Figure 2B). GO enrichment analysis showed genes within M1 were enriched for functions such as regulation of organelle organization, the transforming growth factor (TGF) β signaling pathway, and the cellular catalysis process (Figure 2C). Genes within M3 were enriched for functions related to cell cycle, phosphorylation, and cellular response to cytokine stimulus (Figure 2C). Genes within M4 were enriched for functions related to cell migration, cell motility, and the metabolism process (Figure 2C). No GO term was enriched by M7 and M8. The KEGG pathway enrichment showed the M1 genes were enriched for focal adhesion, protein processing, cell cycle, and a variety of signaling pathways such as Wnt and PI3K-Akt signaling pathways (Figure 2D). The M3 and M4 genes were enriched for some similar pathways as M1, and additional pathways were also enriched by the M3 and M4 genes. For example, the M3 genes were enriched for the Toll-like receptor signaling pathway and the biosynthesis of cofactors and amino acids, while the M4 genes were enriched for signaling pathways regulating pluripotency of stem cells, apoptosis, and the Hippo signaling pathway (Figure 2D). The M7 genes were enriched for the mTOR signaling pathway, while the M8 genes were enriched for tight junction (Figure 2D).

### 3.3. DEGs in RE Cells Treated with GA and VP

**Figure 3:**
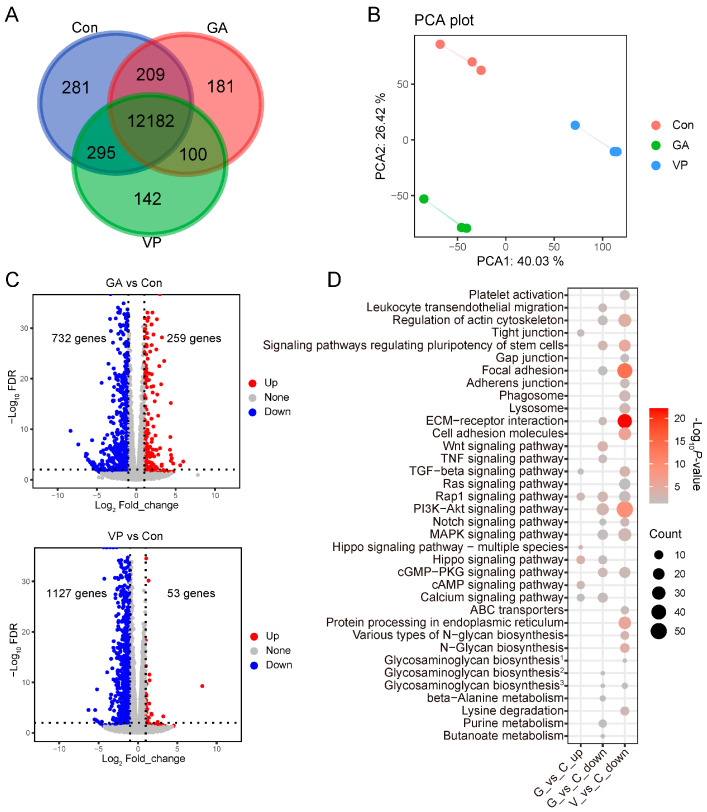
Transcriptomic expression changes in rumen epithelial cells treated with GA-017 (GA) and verteporfin (VP) compared to the control (Con) group. (**A**) Number of genes expressed in common among the GA, VP, and Con groups. (**B**) The difference in transcriptomic profiles among the GA, VP, and Con groups. (**C**) The differentially expressed genes (DEGs) in GA and VP groups when compared to Con. (**D**) KEGG pathways enriched by the DEGs in GA and VP groups. ^1^ Glycosaminoglycan biosynthesis: keratan sulfate. ^2^ Glycosaminoglycan biosynthesis: heparan sulfate/heparin. ^3^ Glycosaminoglycan biosynthesis: chondroitin sulfate/dermatan sulfate.

Although the RE cells in the Con, GA, and VP groups shared most of their expressed genes (Figure 3A), the transcriptomic profiles of these three groups were different from each other (Figure 3B). In the GA group, 259 genes were upregulated and 732 genes were downregulated compared to Con (Figure 3C). These DE genes were enriched for GO terms such as organ development, cell apoptosis, and migration as well as KEGG pathways such as Hippo, calcium, Wnt, and Rap1 signaling pathways (Figure 3D, Tables S3 and S4). In the VP group, 53 genes were upregulated and 1127 genes were downregulated compared to Con (Figure 3C). GO terms such as cellular development, cell differentiation, adhesion, and migration as well as KEGG pathways such as the PI3K-Akt signaling pathway, ECM–receptor interaction, and focal adhesion were enriched by the downregulated genes (Figure 3D and Table S5). No GO term or KEGG pathway was enriched by the upregulated genes of the VP group.

To validate the results of RNA-seq, the expressions of *YAP1*; the upstream regulators of *YAP1*, such as large tumor suppressor kinase 1 and 2 (*LATS1*/*2*) and MOB kinase activator 1A (*MOB1A*); and the downstream TEADs were selected for qPCR analysis. Most of them showed similar changes when compared to Con (Figure S1A). The significant correlation (cor = 0.47, *p*-value < 0.0001) between the results of RNA-seq and qPCR suggested the RNA-seq data were reliable (Figure S1B).

### 3.4. Selected Genes and Pathways Related to RE Cell Viability

**Figure 4:**
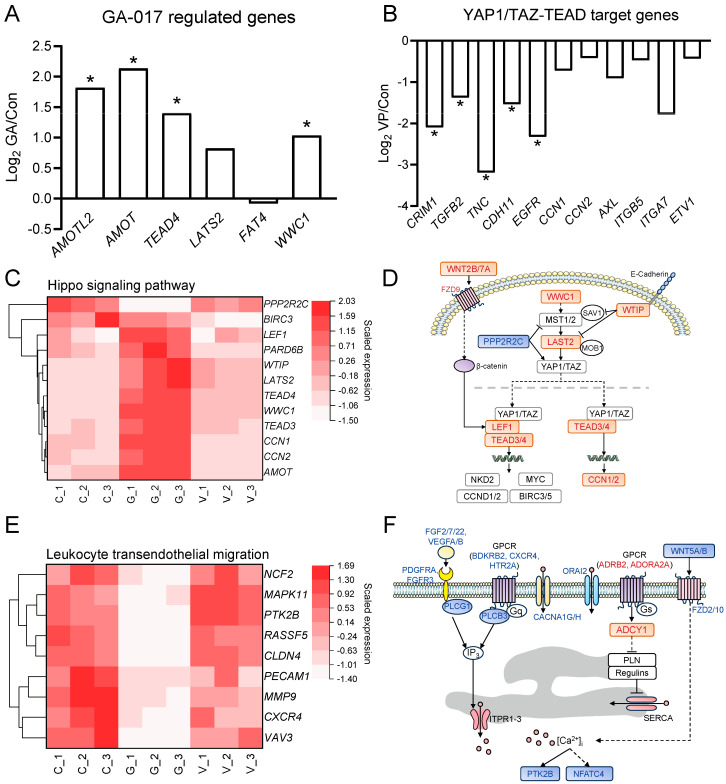
Changes in selected genes and pathways related to rumen epithelial cell viability in cells treated with GA-017 (GA) and verteporfin (VP). (**A**) Changes in GA-related genes in GA-treated cells. (**B**) Changes of YAP1/TAZ target genes in VP-treated cells. (**C**,**E**) The heatmap shows the mRNA expression of genes enriched for the Hippo signaling pathway (**C**) and leukocyte transendothelial migration (**E**). (**D**,**F**) Gene network of Hippo signaling pathway (**D**) and calcium signaling pathway (**F**). Gene names in red represent upregulated genes in the GA group, while those in blue represent downregulated genes in the GA group compared to the control group. * represents differentially expressed genes in GA or VP.

Compared to Con, among the genes regulated by GA as previously reported [12], angiomotin-like 2 (*AMOTL2*), angiomotin (*AMOT*), *TEAD4*, *LATS2*, and WW and C2 domain containing 1 (*WWC1*) were upregulated in the GA group (Figure 4A). Among the YAP1/TAZ-TEAD target genes [26], cysteine-rich transmembrane BMP regulator 1 (*CRIM1*), TGF β2 (*TGFB2*), tenascin C (*TNC*), cadherin 11 (*CDH11*), and epidermal growth factor receptor (*EGFR*) were downregulated in the VP group compared to Con (Figure 4B).

Both the cell viability-related modules and GA-regulated DEGs were enriched for the Hippo and Wnt signaling pathways as well as leukocyte transendothelial migration (Figure 2D and Figure 3D). In the Hippo signaling pathway, *WWC1*, WT1 interacting protein (*WTIP*), *LAST2*, *TEAD3*/*4*, lymphoid enhancer binding factor 1 (*LEF1*), Wnt family members 2B and 7A (*WNT2B*/*7A*), frizzled class receptor 9 (*FZD9*), and cellular communication network factors 1 and 2 (*CCN1*/*2*) were upregulated, and the protein phosphatase 2 regulatory subunit B gamma (*PPP2R2C*) was downregulated in the GA group compared to Con (Figure 4C,D). The downstream genes of the Hippo signaling pathway, such as NKD inhibitor of WNT signaling pathway 2 (*NKD2*), MYC proto-oncogene (*MYC*), cyclin D1 and D2 (*CCND1*/*2*), and baculoviral IAP repeat-containing 5 (*BIRC5*), were numerically increased in the GA group compared to Con (Figure S2A). In the Wnt signaling pathway, genes such as *WNT5A*/*5B*/6/*7B*/*10A*, *FZD2*/*10*, and others were downregulated in the GA group compared to Con (Figure S2B,C). In the leukocyte transendothelial migration, the vav guanine nucleotide exchange factor 3 (*VAV3*), claudin 4 (*CLDN4*), protein tyrosine kinase 2 β (*PTK2B*), Ras association domain family member 5 (*RASSF5*), matrix metallopeptidase 9 (*MMP9*), mitogen-activated protein kinase 11 (*MAPK11*), C-X-C motif chemokine receptor 4 (*CXCR4*), platelet and endothelial cell adhesion molecule 1 (*PECAM1*), and neutrophil cytosolic factor 2 (*NCF2*) were downregulated in the GA group compared to Con (Figure 4E). In addition, the DEGs of the GA group were enriched for the calcium signaling pathway (Figure 3D). The enriched genes, such as calcium voltage-gated channel subunit alpha1 G and H (*CACNA1G*/*H*) and ORAI calcium release-activated calcium modulator 2 (*ORAI2*), were downregulated in the GA group compared to Con (Figure 4F and Figure S1D). Although the DEGs of the VP group were not enriched for the Hippo, Wnt, and calcium signaling pathways, *BIRC3* belonging to the Hippo signaling pathway was downregulated (Figure 4C).

### 3.5. DEGs in RE Cells Treated with SB

**Figure 5:**
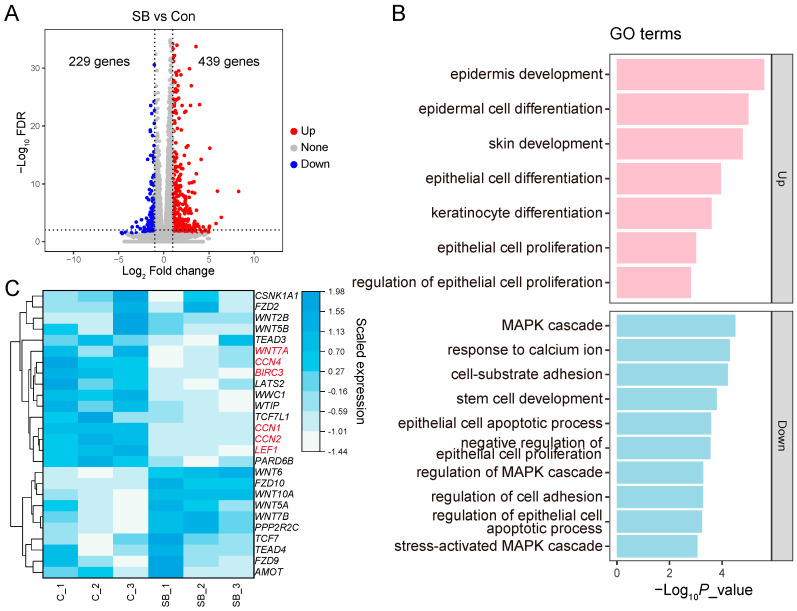
Transcriptomic expression changes in rumen epithelial cells treated with sodium butyrate (SB) compared to the control (Con) group. (**A**) The differentially expressed genes (DEGs) in the SB group compared to Con. (**B**) GO terms related to epithelial development enriched by the DEGs. (**C**) The mRNA expressions of genes involved in Hippo and Wnt signaling pathways. Gene names in red represent DEGs in the SB group compared to Con.

Compared to Con, the cell viability was significantly increased in the RE cells treated with 1 μM or more SB for 24 h (Figure S3). Thus, 1 μM SB and a 24 h incubation time were selected for Experiment 2. In Experiment 2, after filtering out the low-quality reads, 283.92 million high-quality reads were obtained across six samples, with an average of 47.32 ± 1.85 (mean ± SD) million reads per sample (Table S2). The mapping rate of clean reads to the reference genome was 84.88%. Compared to Con, 439 genes were upregulated and 229 genes were downregulated in the SB group (Figure 5A). These genes were enriched for functions related to epithelial development (Figure 5B and Figure S4).

### 3.6. Divergence of Gene Expression in Cells Treated with GA and SB

**Figure 6:**
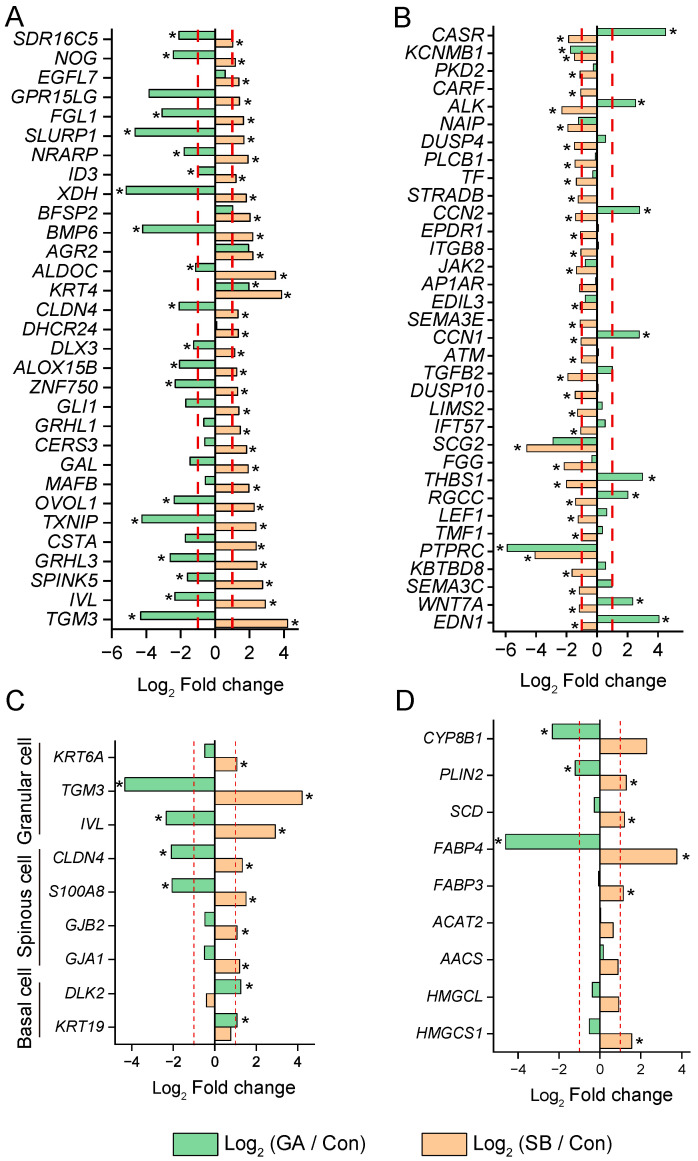
Divergence of gene expression in cells treated with GA-017 (GA) and sodium butyrate (SB). (**A**,**B**) Changes in genes involved in epithelial development in cells treated with GA and SB compared to the control (Con) group. (**C**) Changes of cell marker genes in cells treated with GA and SB compared to Con. (**D**) Changes in genes related to SCFA metabolism in cells treated with GA and SB compared to Con. * represents DEGs in GA and SB compared to Con. The red dash line indicates |Log_2_ Fold change| = 1.

To investigate whether YAP1/TAZ was involved in SB-induced RE cell proliferation, we focused on the genes that changed in Experiment 1 (Figure 5C and Figure S5). Within the Hippo and Wnt signaling pathways, *WNT7A*, *CCN1*/*2*, and *LEF1*, which were upregulated in the GA-treated cells, were downregulated in the SB group compared to Con (Figure 5C). *BIRC3*, which was downregulated in the VP-treated cells, was also downregulated in the SB group compared to Con (Figure 5C). *CCN4*, which was not altered by GA, was downregulated in the SB group (Figure 5C). Other genes involved in the Hippo and Wnt signaling pathways, as well as genes involved in the calcium signaling pathway, remained unchanged in SB-treated cells (Figure 6C and Figure S5).

Then, we focused on the genes related to epithelial development that exhibited changes in Experiment 2 (Figure 6A,B). Figure 6A shows the upregulated genes in SB-treated cells that were enriched for epithelial development, proliferation, and differentiation. However, most of these genes were downregulated in GA-treated cells, such as transglutaminase 3 (*TGM3*), involucrin (*IVL*), serine peptidase inhibitor Kazal type 5 (*SPINK5*), and others. Only keratin 4 (*KRT4*) showed a similar change in cells treated with GA to that in cells treated with SB. Figure 6B shows the downregulated genes in SB-treated cells that were enriched for epithelial cell apoptotic processes, cell adhesion, response to calcium ions, and the MAPK cascade. Among them, genes such as endothelin 1 (*EDN1*), *WNT7A*, and *CCN1*/*2* were upregulated in GA-treated cells. Only protein tyrosine phosphatase receptor type C (*PTPRC*) and potassium calcium-activated channel subfamily M regulatory beta subunit 1 (*KCNMB1*) showed similar changes in cells treated with GA to those in cells treated with SB compared to Con.

The expressions of cell marker genes and genes related to SCFA metabolism are presented in Figure 6C,D, respectively. For the basal cell markers, *KRT19* [27] and delta-like non-canonical Notch ligand 2 (*DLK2*) [28] remained unchanged in SB-treated cells, while they were upregulated in GA-treated cells. For the spinous and granular cell markers [27,29,30], all of them were upregulated in SB-treated cells, while S100 calcium binding protein A8 (*S100A8*), *CLDN4*, *IVL*, and *TGM3* were downregulated in GA-treated cells. Among the genes related to SCFA metabolism, 3-hydroxy-3-methylglutaryl-CoA synthase 1 (*HMGCS1*), fatty acid binding proteins 3 and 4 (*FABP3*/*4*), stearoyl-CoA desaturase (*SCD*), and perilipin 2 (*PLIN2*) were upregulated in the SB-treated cells. In contrast, *FABP4* and *PLIN2* were downregulated in the GA-treated cells, while other genes remained unaffected (Figure 6D). The 5-beta-cholestane-3-alpha,7-alpha-diol-12-alpha-hydroxylase (*CYP8B1*) gene was not changed in the SB-treated cells but was downregulated in the GA-treated cells (Figure 6D).

## 4. Discussion

YAP1/TAZ has been revealed to regulate ovulation in bovines [15], be associated with pregnancy recognition and establishment in ewes [31], and be involved in embryonic development in cows [32]. These studies have opened the prelude to the study of YAP1/TAZ in ruminants. To date, their function in the gastrointestinal development of ruminants is still unclear. In the present study, we used a YAP1/TAZ activator and inhibitor to indirectly assess the potential impact of YAP1/TAZ on RE cell proliferation. Our results provide theoretical guidance on the potential for promoting rumen development through YAP1/TAZ activators.

In the present study, the upregulated GA-related genes and downregulated YAP1/TAZ target genes in RE cells treated with GA or VP, respectively, suggest conserved roles of GA and VP in different types of cells. These results also suggest the suitability of these two small molecules for studying the role of YAP1/TAZ in RE. The results of RE cell viability suggested that RE cell proliferation could be mediated by regulating YAP1/TAZ activation. When treated with the YAP1/TAZ activator, genes involved in Hippo, Wnt, and calcium signaling pathways were altered in RE cells. The Hippo cascade is a primary regulator of YAP1/TAZ in a variety of physiological processes [33]. Ca^2+^ has been identified as a potential second messenger closely associated with the regulation of YAP1/TAZ [34]. In metastatic melanoma cells, YAP1/TAZ activity could be increased by reducing Ca^2+^ influx [35]. In the present study, the decreased expression of *CACNA1G*/*H* and *ORAI2* in the RE cells could lead to a reduction in store-operated calcium entry [36,37], which may be further associated with YAP1/TAZ activation. On the other hand, upon YAP1/TAZ activation, genes within the Wnt signaling pathway were suppressed in the RE cells, which is similar to the changes in YAP1-dependent reprogramming of *LGR5*^+^ stem cells during intestinal regeneration and cancer [38]. Further investigation is needed to understand the interaction between calcium and YAP1/TAZ as well as the interaction between the Wnt signaling pathway and YAP1/TAZ during RE cell proliferation. In addition, YAP1/TAZ target genes, such as *CCN1*/*2* [39], *CCND1*/*2* [40,41], *MYC* [42], and *BIRC5* [43], are involved in promoting the proliferation of stratified squamous epithelial cells. Their increase might be responsible for the proliferation of RE cells induced by GA. When treated with the YAP1/TAZ inhibitor, the decrease in cell proliferation was accompanied by a decrease in gene expression of the YAP1/TAZ target gene *BIRC3*. *BIRC3* is a member of the inhibitor of apoptosis (IAP) family, which can promote cell proliferation during tumorigenesis [44]. Its expression has been reported to be upregulated in squamous cell cancers, such as esophagus cancer [45]. The decreased expression of *BIRC3* in the VP-treated cells suggests its role in regulating RE cell proliferation. These results suggest that YAP1/TAZ could be potential targets for regulating RE cell proliferation. Activating YAP1/TAZ promotes RE cell proliferation by increasing *CCN1*/*2*, *CCND1*/*2*, *MYC*, and *BIRC5*, while inhibiting YAP1/TAZ suppresses RE cell proliferation by decreasing *BIRC3*.

YAP1 is reported to play a critical role in regulating innate and adaptive immunity. For example, in the Kupffer cells, activation of YAP1 enhanced the production of proinflammatory cytokines and promoted the development of liver inflammation [46]. In endothelial cells, enhanced YAP1 expression could promote the expression of inflammatory genes in atherosclerotic plaques [47]. In the present study, we observed a negative relationship between the immune-related pathway “leukocyte transendothelial migration” and RE cell proliferation. Among the downregulated DEGs of GA that were enriched for this pathway, *PTK2B* was significantly upregulated in the inflamed mucosa of patients with ulcerative colitis [48]. The expression of *MMP9* in the intestinal epithelium was positively correlated with the level of proinflammatory cytokines [49]. A higher expression of *MMP9* was also observed in the inflamed RE of goats that was induced by a high-concentrate diet [50]. The increased expression of *MAPK11* was associated with the enhancement of liver inflammation [51]. The *CXCR4*–*CXCL12* axis has been linked to the recruitment of immune cells in colorectal cancer [52]. The expression of *NCF2* is increased in squamous cervical cancer. These genes show a positive relationship with inflammation. The decreased expression of these genes suggests a low risk of inflammation under GA treatment. Thus, activating YAP1/TAZ might be a feasible method for promoting RE proliferation with a low risk of inflammation. However, proper nutrient activators of YAP1/TAZ should be filtered, and an in vivo study is needed to confirm the effect and safety of the filtered nutrient.

It seems that SB cannot regulate YAP1/TAZ, as indicated by the differential gene expressions between GA- and SB-treated cells compared to Con, such as the YAP1/TAZ target genes as well as genes related to the Hippo, Wnt, and calcium signaling pathways; epithelial cell development, proliferation, differentiation, and apoptosis; and SCFA metabolism. Although both GA and SB promoted the proliferation of RE cells, the distinct changes in the expression of cell markers might suggest an increase in basal cells in the GA group but an increase in spinous and granular cells in the SB group. On the other hand, the *GJA1*, *GJB2*, and *S100A8* highly expressed cells were spinous cells involved in SCFA metabolism [29]. Their increases in SB-treated cells were consistent with the increases in genes related to SCFA metabolism. These changes were not observed in cells treated with GA. Thus, GA might enhance the proliferation of RE cells by increasing basal cells, while SB might enhance RE cell proliferation and SCFA metabolism by increasing spinous and granular cells. These changes in cell numbers for each cell type require further confirmation.

## 5. Conclusions

In summary, YAP1/TAZ could be potential targets for regulating RE cell proliferation, but not for SCFA metabolism. The Hippo, Wnt, and calcium signaling pathways were altered in cells upon the regulation of YAP1/TAZ. Upon YAP1/TAZ activation, *CCN1*/*2*, *CCND1*/*2*, *MYC*, and *BIRC5* were upregulated to promote RE cell proliferation. However, when YAP1/TAZ was inhibited, *BIRC3* was decreased to suppress RE cell proliferation. SB is not a YAP1/TAZ activator. YAP1/TAZ activation might enhance the proliferation of RE cells by increasing basal cells, while SB might enhance RE cell proliferation and SCFA metabolism by increasing spinous and granular cells. Thus, nutrients that regulate YAP1/TAZ need to be filtered for future use in animal feeding. These findings broaden our understanding of the role of YAP1/TAZ and their regulators in RE and offer a potential target for promoting rumen development. However, our present study failed to find a specific antibody for YAP1/TAZ to indicate the activation of YAP1/TAZ at the protein level. The direct evidence of YAP1/TAZ activation was not provided. We did not verify the interaction between YAP1/TAZ and other pathways, such as Wnt and calcium signaling pathways. Further study is needed to confirm the interactions and determine the significance of YAP1/TAZ in RE cell proliferation. Furthermore, the present study used cultured RE cells. Animal trials are needed to confirm the function of YAP1/TAZ in vivo.

## Data Availability

The RNA sequencing data have been uploaded to the Gene Expression Omnibus (https://www.ncbi.nlm.nih.gov/geo/) with the access numbers GSE252747 and GSE252749.

## Supplementary Materials

The following supporting information can be downloaded at https://www.mdpi.com/article/10.3390/ani14060922/s1, Figure S1: Quantitative PCR (qPCR) verification of transcriptomic expression of YAP1-related genes; Figure S2: Changes in genes involved in selected pathways in cells treated with GA-017 (GA) and verteporfin (VP) compared to the control (Con) group; Figure S3: Effect of different concentrations of sodium butyrate (SB) (0, 0.25, 0.5, 1, 2.5, 5, 10, 25, and 50 µM) on the viability of rumen epithelial cells at different incubation times (4, 8, 12, 24, and 48 h); Figure S4: Function enrichment of differentially expressed genes (DEGs) in cells treated with sodium butyrate (SB) compared to the control group; Figure S5: Heatmap shows the mRNA expression of genes involved in the calcium signaling pathway in cells treated with sodium butyrate (SB) or not (Con); Table S1: Information on the primers used for gene expression validation; Table S2: Sequence quality of transcriptomic data generated from Experiments 1 and 2; Table S3: GO terms enriched by upregulated genes induced by GA-017 when compared to the Con group; Table S4: GO terms enriched by downregulated genes induced by GA-017 when compared to the Con group; Table S5: GO terms enriched by downregulated genes induced by verteporfin when compared to the Con group.
