# Supplementary material for: YAP1/TAZ Mediates Rumen Epithelial Cell Proliferation but Not Short-Chain Fatty Acid Metabolism In Vitro
Source: Animals (Basel). 2024 Mar 17;14(6):922. doi: 10.3390/ani14060922 (PMC10967441; doi:10.3390/ani14060922)
Supplement: Supplementary file 1 [file animals-14-00922-s001.zip › supplement_figures.pdf]

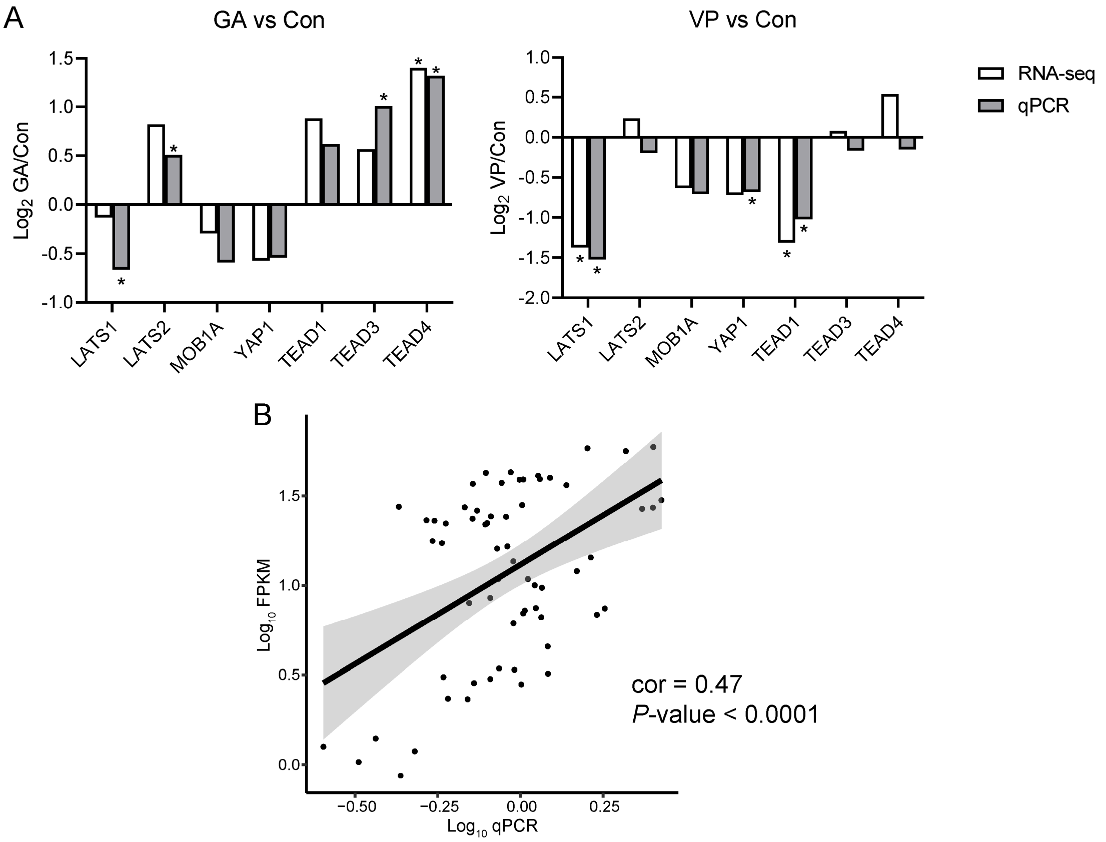

Figure S1. Quantitative PCR (qPCR) verification of transcriptomic expression of YAP1 related genes. (**A**) Comparison between the results of RNA-seq and qPCR. * represents DE gene for RNA-seq and represents *P*-value < 0.05 for qPCR. (**B**) Correlation between the results of RNA-seq and qPCR.

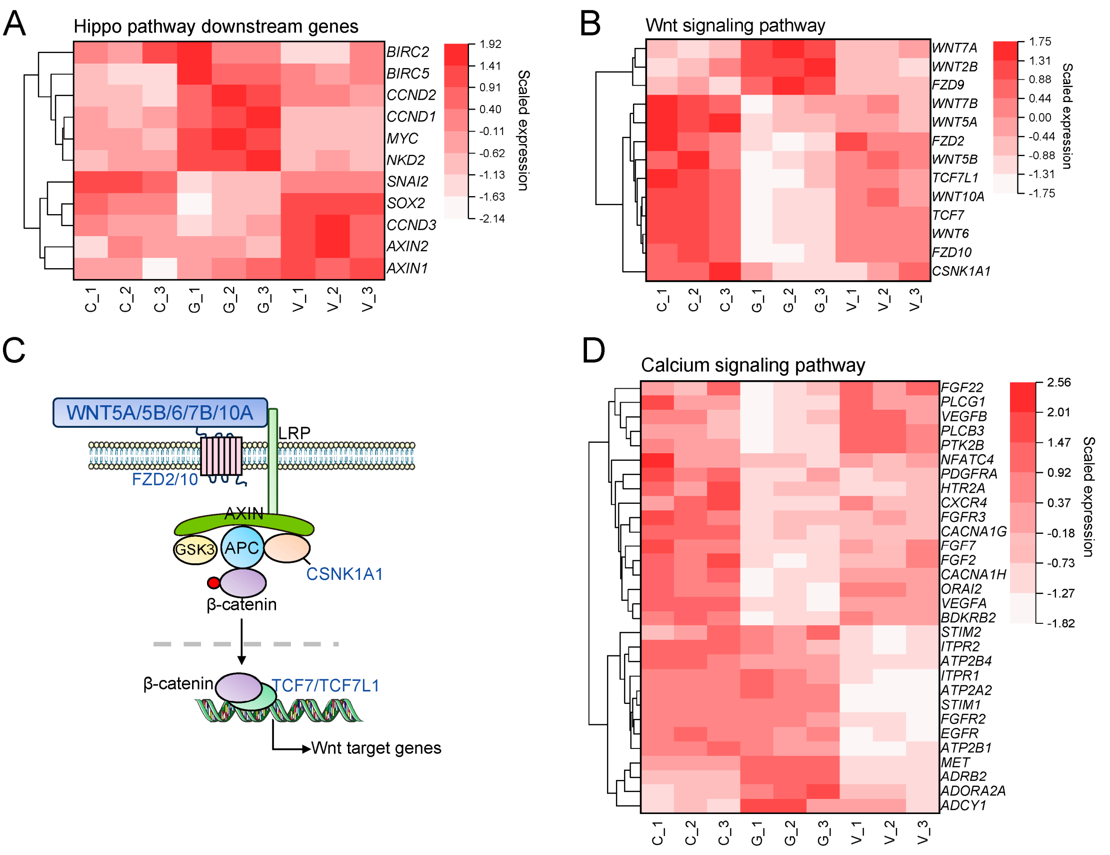

Figure S2. Changes of genes involved in selected pathways in cells treated with GA-017 (GA) and verteporfin (VP) compared to control (Con) group. (**A**) Heatmap shows the mRNA expression of the downstream genes of Hippo signaling pathway. (**B**) Heatmap shows the mRNA expression of genes involved in Wnt signaling pathway. (**C**) Gene network of Wnt signaling pathway. Gene names in bule represent downregulated genes in GA group compared to Con. (**D**) Heatmap shows the mRNA expression of genes involved in calcium signaling pathway. C, G, and V represent samples from Con, GA, and VP, respectively.

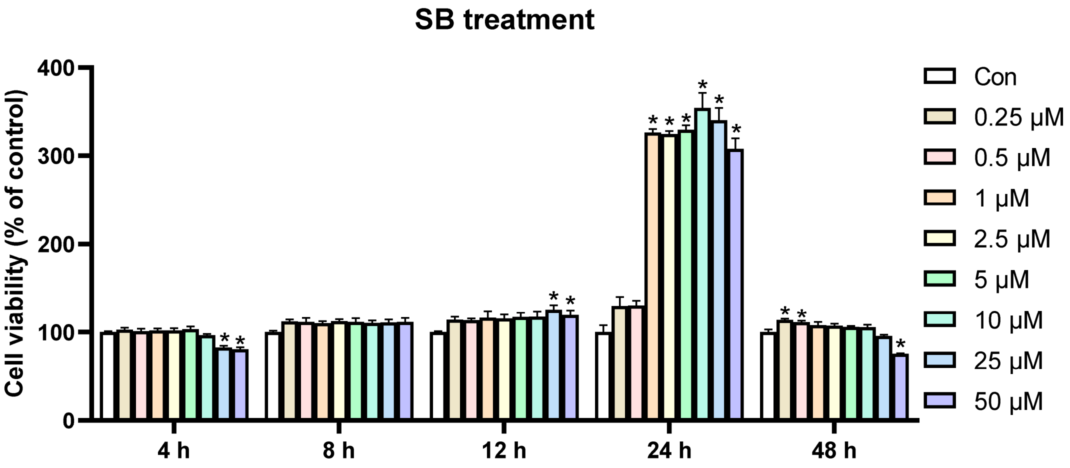

Figure S3. Effect of different concentrations of sodium butyrate (SB) (0, 0.25, 0.5, 1, 2.5, 5, 10, 25, and 50 μM) on the viability of rumen epithelial cells at different incubation time (4, 8, 12, 24, and 48 h).

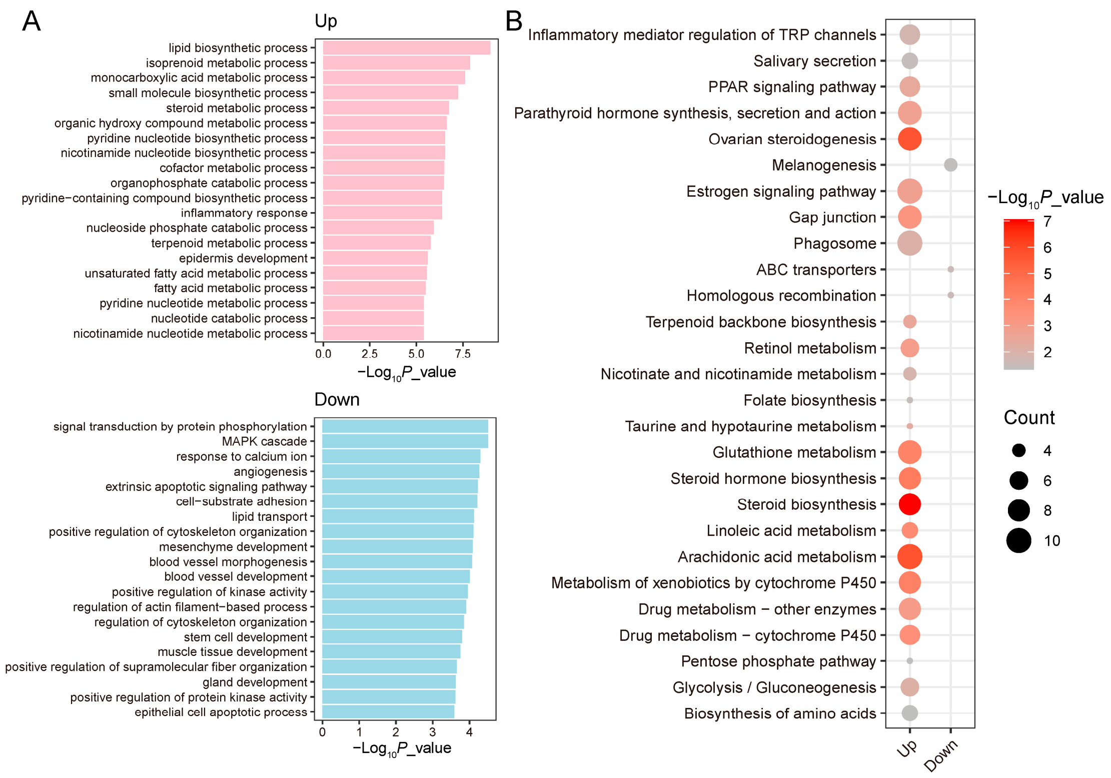

Figure S4. Function enrichment of differentially expressed genes (DEGs) in cells treated with sodium butyrate (SB) compared to control group. (**A**) Top 20 GO terms enriched by DEGs. (**B**) KEGG pathways enriched by DEGs.

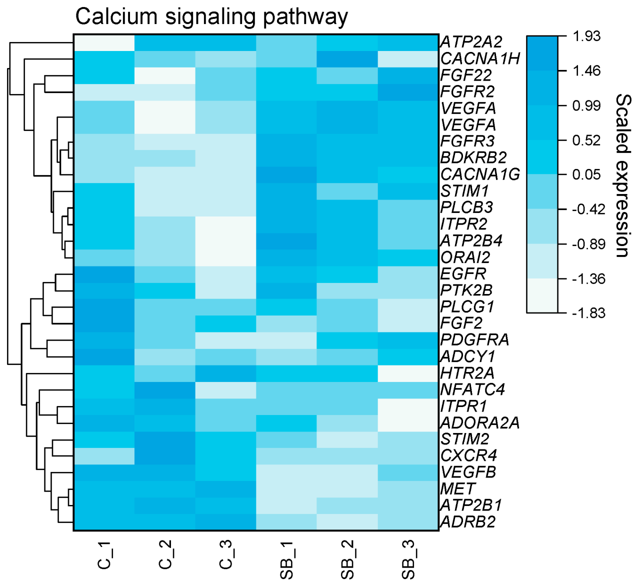

Figure S5. Heatmap shows the mRNA expression of genes involved in calcium signaling pathway in cells treated with sodium butyrate (SB) or not (Con).
